# Prevalence, antimicrobial resistance and biofilm forming ability of *Escherichia coli* in milk, animal handlers and slurry samples from dairy herds of Punjab, India

**DOI:** 10.3389/fvets.2025.1553468

**Published:** 2025-06-17

**Authors:** Deepthi Vijay, Jasbir Singh Bedi, Pankaj Dhaka, Randhir Singh, Jaswinder Singh, Anil Kumar Arora, Jatinder Paul Singh Gill

**Affiliations:** ^1^Centre for One Health, College of Veterinary Science, Guru Angad Dev Veterinary and Animal Sciences University, Ludhiana, India; ^2^Department of Veterinary and Animal Husbandry Extension Education, College of Veterinary Science, Guru Angad Dev Veterinary and Animal Sciences University, Ludhiana, India; ^3^Department of Veterinary Microbiology, College of Veterinary Science, Guru Angad Dev Veterinary and Animal Sciences University, Ludhiana, India

**Keywords:** antimicrobial resistance genes, biofilms, dairy, *E. coli*, India, milk

## Abstract

Antimicrobial-resistant *Escherichia coli* serves as an indicator for monitoring the occurrence of antimicrobial resistance (AMR) at the human, animal, and environmental interface. The present study employs a ‘One Health’ framework to determine the prevalence, phenotypic and genotypic characterisation of the AMR profile and biofilm forming ability of *E. coli* isolates from milk, hand swabs and slurry samples of 405 dairy herds in Punjab, India. An overall prevalence of *E. coli* was 34.3% (139/405) (95% CI: 0.30 to 0.39) from pooled milk samples, 9.1% (37/405) (95% CI: 0.06 to 0.12) from the hand swabs of animal handlers and 64.4% (261/405) (95% CI: 0.60 to 0.69) in the slurry samples. Multidrug resistance (resistance to 3 or more classes of antimicrobials) was exhibited by 24.4% (34/139) of *E. coli* isolates from milk, 40.5% (15/37) from hand swabs, 60.5% (158/261) from slurry samples. Moreover, of the *E. coli* isolates, 11.51% (16/139) from milk, 24.32% (09/37) from hand swabs and 31.42% (82/261) from slurry samples were resistant to 5 or more antimicrobial classes. On molecular characterisation, 19.4% (27/139) of *E. coli* isolates from milk, 37.8% (14/37) from hand swabs, and 33.3% (87/261) from slurry samples harboured various genes. Principal component analysis and Shannon-Wiener diversity indices highlighted varying *β*-lactamase (ESBL/AmpC β-lactamase) gene distributions across samples, with milk exhibiting the highest diversity. Logistic regression analysis revealed a significant protective effect of milk hygiene scores against *E. coli* occurrence (OR = 0.18; 95% CI: 0.13–0.26, *p* < 0.001), while linear regression demonstrated a significant negative association between milk hygiene scores and the Multiple Antibiotic Resistance (MAR) index (*p* = 0.02). Biofilm assays revealed that 19.2% of isolates were strong biofilm formers, with a strong association (*p* < 0.01) between biofilm formation potential and MAR index. The multidrug-resistant (MDR) isolates were predominantly moderate biofilm producers, with 23.5% (milk), 20% (hand swabs), and 24.1% (slurry) classified as strong biofilm formers. The study findings underscore the need for One Health-integrated strategies to holistically address AMR challenges at the dairy-environment interface.

## 1 Introduction

The escalating arms race between antimicrobial agents and resistant pathogens is tipping alarmingly in favor of the latter, leading to the emergence of multidrug-resistant (MDR), extensively drug-resistant (XDR), and totally drug-resistant (TDR) pathogens (1). Antimicrobial resistance (AMR) has emerged as a pressing global health challenge, linking its spread to the overuse and misuse of antimicrobials in human healthcare, animal husbandry, and agricultural practices (2). The rise of superbugs such as extended-spectrum *β*-lactamase (ESBL) and AmpC β-lactamase producing *Enterobacteriaceae*, which are included in the World Health Organization’s priority list of bacterial pathogens, is of particular concern particularly in the context of food producing animals, as these opportunistic pathogens thrive in various ecological niches, including humans, animals, and the environment (3). This complex interplay underscores the principles of the One Health approach, a collaborative and multisectoral framework that recognizes the intrinsic connections between human, animal, and environmental health. Within this context, livestock serve as important reservoirs for the selection and dissemination of AMR bacteria, thereby posing significant risks to human and animal health as well as food safety (4). In South Asian countries like India, the inappropriate use of antibiotics has further exacerbated the issue, with reports of resistance to last-resort antibiotics such as carbapenems and colistin (5). The failure to timely identify drug resistant pathogens harboring genes like ESBLs delays appropriate antimicrobial therapy, often leading to poor clinical outcomes, increased morbidity, and mortality. This risk is particularly pronounced in low- and middle-income countries (LMICs), where interconnected human-animal-environment systems accelerate the bidirectional exchange of resistant strains (6, 7).

Antimicrobial-resistant *Escherichia coli* is a significant global health concern due to its ability to harbor and disseminate diverse resistance genes, often facilitated by horizontal gene transfer. In dairy systems, *Enterobacteriaceae* such as *E. coli* serve as useful indicators in surveillance studies due to their ability to acquire AMR genes and as general indicators of hygiene and contamination (8, 9). Further, within dairy farming environments, the intestinal microbiota of food-producing animals serves as a source of antimicrobial-resistant commensals, facilitating the spread of resistant bacteria and AMR genes through the food chain (10). Thereby dairy farms could serve as reservoir of resistance posing significant threats to human health by enabling colonization and infection, which can result in severe outbreaks or silent enteric colonization, particularly among vulnerable populations. Therefore, addressing the role of drug-resistant pathogens in dairy farming systems is essential to understand and mitigate the broader implications of AMR in human, animal, and environmental health.

Previous studies observed the prevalence of multidrug-resistant *E. coli* in raw milk as 34.3% in China, 20% in Egypt, and 25% in Ethiopia (11–13). These findings emphasize the role of raw milk as a possible vehicle for transmitting resistant pathogens to humans. Furthermore, occupational exposure among dairy handlers exacerbates the risk of colonization and infection (14, 15). Manure from livestock farms is a significant source of mobile genetic elements, which contribute to the dissemination of resistance genes in agricultural soils (16, 17). The prevalence of ESBL and AmpC *β*-lactamase genes in dairy farm waste and their persistence in solid manure and wastewater highlight their resilience and potential for environmental dissemination (18). Further, *β*-lactamase genes in *E. coli* from farm environment has shown diverse genotypes and plasmid-mediated gene transfer, underlining the complexity of its epidemiology in dairy farms (19).

Advancements in molecular diagnostics have enhanced our understanding of the genetic mechanisms driving AMR in *E. coli* from dairy ecosystems. Commonly identified genes include those encoding β-lactamases (*blaTEM*, *blaSHV*, *blaCTX-M*), tetracycline resistance (*tetA*, *tetB*), and sulfonamide resistance (*sul1*, *sul2*) (15, 20). Of particular concern are plasmid-mediated resistance elements, which enable the rapid horizontal transfer of resistance determinants across microbial populations (21). Phylogenetic analyses reveal genetic clustering of resistance genes from human, animal, and environmental reservoirs, demonstrating the interconnectedness of resistance dynamics globally (22). Biofilm formation further exacerbates the AMR issue by enhancing bacterial tolerance and resistance (23, 24). Resistant biofilms, which are likely to form in dairy farm settings due to the constant presence of moisture, organic matter, and bacteria, pose a significant challenge in AMR management, as they shield bacteria from antimicrobial agents and promote the horizontal gene transfer (HGT) of antimicrobial resistance genes (ARGs), thereby enhancing the spread of resistance (25, 26).

There are a limited number of studies focusing on the dissemination of multidrug resistant pathogens including *β*-lactamase (ESBL/AmpC β-lactamase) producing bacteria in the dairy farm environment in India, highlighting a critical gap in understanding their role in antimicrobial resistance within this sector (27, 28). Considering the importance of assessing *E. coli* as an indicator for AMR through surveillance, the present study investigates the prevalence, AMR profiles, genetic determinants, and biofilm forming ability of *E. coli* in dairy farms of Punjab, India.

## 2 Materials and methods

### 2.1 Study design and sample collection

The cross-sectional study was conducted from December 2020 to October 2021 across dairy farms in Punjab, India. The required sample size of 384 dairy herds was calculated to estimate the prevalence of *E. coli* with 95% confidence and 5% absolute precision, assuming 50% expected prevalence. A total of 405 farms were ultimately enrolled in the study from Punjab’s three agro-climatic zones, i.e., central plains, undulating plains, and western zone, which were stratified, and from each zone, two districts were randomly selected. Within each district, one tehsil and five villages were randomly chosen, resulting in 30 villages across six districts. The farm selection in the study was guided by proportional allocation based on regional milch animal populations, with deliberate inclusion of both household-level and commercial farms to enhance representativeness and reduce selection bias. Sampling was proportional to the regional milch animal population (29), with 215 farms sampled from the central plains, 100 from the undulating plains, and 90 from the western zone. Proportionately, 341 household-level herds (<10 animals) and 64 commercial farms (≥10 animals) were enrolled. Although the study employed a stratified multistage sampling design with random selection of districts, tehsils, and villages across Punjab’s three agro-climatic zones, the final farm enrolment within villages was based on farmer willingness, and convenience sampling was employed where random selections were declined.

From each farm, pooled milk, handler hand swabs, and herd slurry samples were collected. Thereby, a total of 1,215 samples comprising of pooled milk samples (*n* = 405), hand swabs (*n* = 405), and slurry samples (*n* = 405) were collected. Raw milk was aseptically pooled, hand swabs were taken with sterile pre-moistened swabs, and slurry samples were collected from three distinct herd locations from each farm. Hand swabs were primarily collected from the main milker responsible for daily milking activities on each farm. In household-level farms, one individual typically handled milking, and a single hand swab was collected. In large commercial farms with multiple milkers, swabs were taken from each milker’s palm and pooled into a single tube to represent the collective hygiene status of the farm’s milking personnel. During the farm visits, the management practices, specifically related to clean milk production and hygiene were recorded. All samples were transported under aseptic conditions and processed immediately or stored at 4°C for up to 24 h.

### 2.2 Isolation and identification of *E. coli*

Enrichment of the samples was performed in brain heart infusion (BHI) broth, followed by selective plating on Eosin Methylene Blue (EMB) agar as per the standard procedure (30, 31). Presumptive *E. coli* colonies were confirmed biochemically using Enterobacteriaceae Identification Test Kits and molecular characterization was carried out by targeting the *ecp* gene through polymerase chain reaction (PCR), as per standardized protocol (32).

### 2.3 Phenotypic and genotypic characterization of antibiotic resistance

The Kirby-Bauer disk diffusion method on Mueller Hinton agar was used to assess susceptibility to various antibiotics. The selection of antibiotics was determined in consultation with veterinary academicians and field experts to focus on those commonly used in the region. A total of 15 antibiotics across various classes namely Quinolones (Ciprofloxacin), Cephalosporins (Cefazolin, Cefuroxime, Ceftriaxone, Ceftazidime, Cefepime), Macrolides (Erythromycin), Aminoglycosides (Gentamicin), Aminopenicilins (Ampicillin, Amoxicillin-Clavulanic acid), Tetracycline, Sulphonamides (Sulpha-TMP (Co-trimoxazole)), Carbapenems (Imipenem and Meropenem) and Monobactams (Aztreonam) was used. The zone diameters were interpreted per (33), and quality was ensured using *E. coli* ATCC 25922 as control. The ESBL production was assessed using cephalosporin-clavulanic acid synergy testing. The multidrug resistance index (MAR) was calculated as per Krumperman (34) as the ratio of the number of antibiotics displaying resistance to the total number of antibiotics tested, and the isolates with MAR more than 0.2 were classified as high risk.

Further, the phenotypic resistant isolates were subjected to characterization of resistance genes, *viz., blaTEM, blaSHV, blaOXA-1, blaAmpC, blaCTXM, tetA, tetB, qnrA, qnrB, qnrS, sul1, sul2, ermB, ermC, and aacA-aphD* using PCR assay. The details of the used primers and reference for the used protocol is provided in Table 1.

**Table 1:** Details of primer sequences of antimicrobial resistance genes for *E. coli*.

**Target gene**	**Primer sequence**	**Amplified product size**	**Annealing temperature**	**Study reference**
*blaTEM*	F- ATGAGTATTCAACATTTCCG	867 bp	52 °C	Bhattacharjee et al. (65)
	R- CTGACAGTTACCAATGCTTA			
*blaSHV*	F- TCGCCTGTGTATTATCTCCC	768 bp	54 °C	Maynard et al. (66)
	R- CGCAGATAAATCACCACAATG			
*blaOXA-1*	F- GCAGCGCCAGTGCATCAAC	198 bp	60 °C	
	R- CCGCATCAAATGCCATAAGTG			
*blaAmpC*	F- CCCCGCTTATAGAGCAACAA	631 bp	57 °C	Shahid et al. (67)
	R- TCAATGGTCGACTTCACACC			
*blaCTXM*	F- CAATGTGCAGCACCAAGTAA	540 bp	60- 65°C^a^	Dutta et al. (68)
	R- CGCGATATCGTTGGTGGTG			
*qnrA*	F- ATT TCTCACGCCAGGATTTG	516 bp	58 °C	Ciesielczuk et al. (69)
	R- GATCGGCAAAGGTTAGGTCA			
*qnrB*	F- GATCGTGAAAGCCAGAAAGG	476 bp	58 °C	
	R- ATGAGCAACGATGCCTGGTA			
*qnrS*	F- GCAAGTTCATTGAACAGGGT	428 bp	58 °C	
	R- TCTAAACCGTCGAGTTCGGCG			
*tet(A)*	F- GTGAAACCCAACATACCCC	888 bp	60 °C	Maynard et al. (66)
	R- GAAGGCAAGCAGGATGTAG			
*tet(B)*	F- CCTTATCATGCCAGTCTTGC	774 bp	60 °C	
	R- ACTGCCGTTTTTTCGCC			
*sul 1*	F- TTCGGCATTCTGAATCTCAC	822 bp	52 °C	Titilawo et al. (70)
	R- ATGATCTAACCCTCGGTCTC			
*sul 2*	F- CGGCATCGTCAACATAACC	722 bp	52 °C	Sáenz et al. (71)
	R- GTGTGCGGATGAAGTCAG			
*ermB*	F- CTATCTGATTGTTGAAGAAGGAT	142 bp	55 °C	Martineau et al. (72)
	R- GTTTACTCTTGGTTTAGGATGAA			
*ermC*	F- AAT CGT CAA TTC CTG CAT GT	299 bp	55 °C	Strommenger et al. (73)
	R- TAA TCG TGG AAT ACG GGT TTG			
*aacA-aphD*	F- TAA TCC AAG AGC AAT AAG GGC	227 bp	55 °C	
	R- GCC ACA CTA TCA TAA CCA CTA			

a The PCR protocol has been modified as: 95°C 5 m/95°C 30s—65°C 30s—72°C 30s (5 cycles) 95°C 30s—62°C 30s—72°C 30s (10 cycles)/95°C 30s—60°C 30s—72°C 30s (15 cycles)/95°C 30s—58 °C 30s—72°C 30s (15 cycles)/72°C 7 m.

### 2.4 Biofilm formation assay

The recovered *E. coli* isolates were subjected to 96-well crystal violet microtiter plate biofilm assay as described by Wakimoto et al. (35), with slight modifications (32). Briefly, overnight cultures were resuspended in 1,000 μL of Luria-Bertani (LB) broth and incubated for 18 h at 37°C. The bacterial suspension was adjusted to 0.5 McFarland units and subsequently diluted 1:100 in LB broth supplemented with 1% glucose. For the assay, 200 μL of the diluted suspension was inoculated into each well of a 96-well polystyrene microtiter plate, while negative controls contained only the culture medium. All tests were conducted in triplicate to ensure reproducibility.

Following incubation, the crystal violet staining procedure was performed. Each standardized bacterial suspension (10^7^–10^8^ CFU/mL) was incubated in the plates at 37°C for 48 h under static conditions. After incubation, wells were gently washed with phosphate-buffered saline (PBS, pH 7.2), fixed with 200 μL methanol for 15 min, and stained with 200 μL of 2% crystal violet solution. Excess stain was removed by washing, and the plates were air-dried. Subsequently, 200 μL of 33% peracetic acid was added to each well to solubilize the stained biofilm. The quantification of biofilm formation of the isolates was done by measuring the absorbance (OD) values at 570 nm. The results were interpreted based on the ODc (control), and the isolates were classified into four biofilm formers categories: (a) non-biofilm former: OD of the test isolate ≤ ODc; (b) weak: OD of the test isolate between ODc and 2 ODc; (c) moderate: OD of the test isolate between 2 ODc and 4 ODc; and (d) strong: OD of the test isolate >4 ODc.

### 2.5 Statistical analysis

The primary hypotheses tested in this study were: (i) there is agreement between phenotypic and genotypic detection of ESBL-producing *E. coli* isolates; (ii) milk hygiene scores are associated with both prevalence of *E. coli* on dairy farms and MAR index among *E. coli* isolates; (iii) herd size is associated with *E. coli* prevalence and MAR index among *E. coli* isolates; and (iv) the degree of biofilm formation in *E. coli* isolates is associated with their antimicrobial resistance profiles, as measured by MAR index values.

The agreement between phenotypic and genotypic detection of ESBL-producing *E. coli* isolates across milk, hand swabs, and dairy herd slurry was assessed using Cohen’s Kappa coefficient. The Kappa values in the range of 0.41–0.60 were interpreted as indicating ‘moderate agreement’, values from 0.61–0.80 as ‘substantial agreement’, and values from 0.81–1.0 as ‘almost perfect agreement’.

The prevalence of *β*-lactamase (ESBL/AmpC β-lactamase) genes were analyzed across sample sources to evaluate clustering patterns. Principal component analysis (PCA) was performed to identify major patterns and reduce dimensionality in the dataset. Resistance gene data were standardized using Z-scores prior to PCA. Components with eigen values >1 were retained, and their scores (PCA1 and PCA2) were saved as new variables for visualization. A scatter plot was generated using PCA1 (X-axis) and PCA2 (Y-axis) to represent clustering patterns by sample type (milk, hand swabs, and slurry). Also, Shannon-Wiener Diversity Index (H′) was calculated to assess the diversity and distribution of the β-lactamase (ESBL/AmpC β-lactamase) genes (*blaSHV, blaTEM*, *blaCTX-M*, *blaAmpC*, and *blaOXA-1*) across three sample sources: milk, hand swabs, and dairy herd slurry. The calculations were based on the absolute counts of isolates carrying each resistance gene within each sample source. The index was calculated using the formula:


$$H\prime =-\sum (p_{i}\times \ln\;p_{i}).$$

where, H′ value indicate diversity and *p_i_* represents the proportion of each resistance gene relative to the total resistance genes in each sample type.

The association between herd size, *E. coli* prevalence, milk hygiene score, and MAR index of dairy farms was evaluated. A farm was considered *E. coli* positive if any of the samples (raw milk, hand swab, or slurry) tested positive for *E. coli*. The milk hygiene score was derived from five components, each scored on a scale of 1 (performed) or 0 (not performed), resulting in a total score range of 0 to 5. The components included: (1) cleaning of milk equipment; (2) cleaning of milkers’ hands before and after milking; (3) cleaning or dipping of the udder before milking, (4) proper disposal of dung from the milking shed as soon as possible; and (5) regular testing for mastitis and segregation of infected animals. The MAR index was calculated as the proportion of antibiotics to which *E. coli* isolates from each farm were resistant. For each farm, the highest MAR index observed among its source samples (milk, slurry, or hand swab) was recorded.

Further the statistical analysis was conducted using logistic regression to assess the effect of herd size and milk hygiene score on *E. coli* occurrence. The odds ratios (OR) were calculated, with a 95% confidence interval (CI), to quantify the strength of association. A linear regression was employed to evaluate the relationship between herd size, milk hygiene score, and MAR index. Regression coefficients (*β*) with their corresponding 95% CI and *p*-values were assessed. A *p*-value < 0.05 was considered statistically significant.

The association between biofilm formation categories (strong, moderate, weak, and non-biofilm former) and MAR index values in *E. coli* isolates has been assessed using Chi-Square test for independence. For each farm, the highest category of biofilm formation observed among its source samples (milk, slurry, or hand swab) was recorded. Also, a box plot was drafted to illustrate the distribution of MAR index values across the biofilm formation categories. The data recording and descriptive statistical analysis was performed using Microsoft Excel, while regression analysis and PCA was carried out using IBM SPSS version 26.0.

## 3 Results

### 3.1 Isolation and identification of *E. coli*

Of the total of 405 milk samples collected from the dairy farms, 34.3% (139/405) (95% CI: 0.30 to 0.39) were found positive for *E. coli* after biochemical as well as molecular characterization using the ‘*ecp*’ gene. An overall prevalence of 9.1% (37/405) (95% CI: 0.06 to 0.12) of *E. coli* was found in the hand swabs of animal handlers. A total of 261 *E. coli* isolates accounting for a prevalence of 64.4% (95% CI: 0.60 to 0.69) were isolated from the dairy herd slurry samples.

### 3.2 Phenotypic and genotypic characterization of AMR

#### 3.2.1 *E. coli* isolates recovered from bovine milk samples

**Figure 1:**
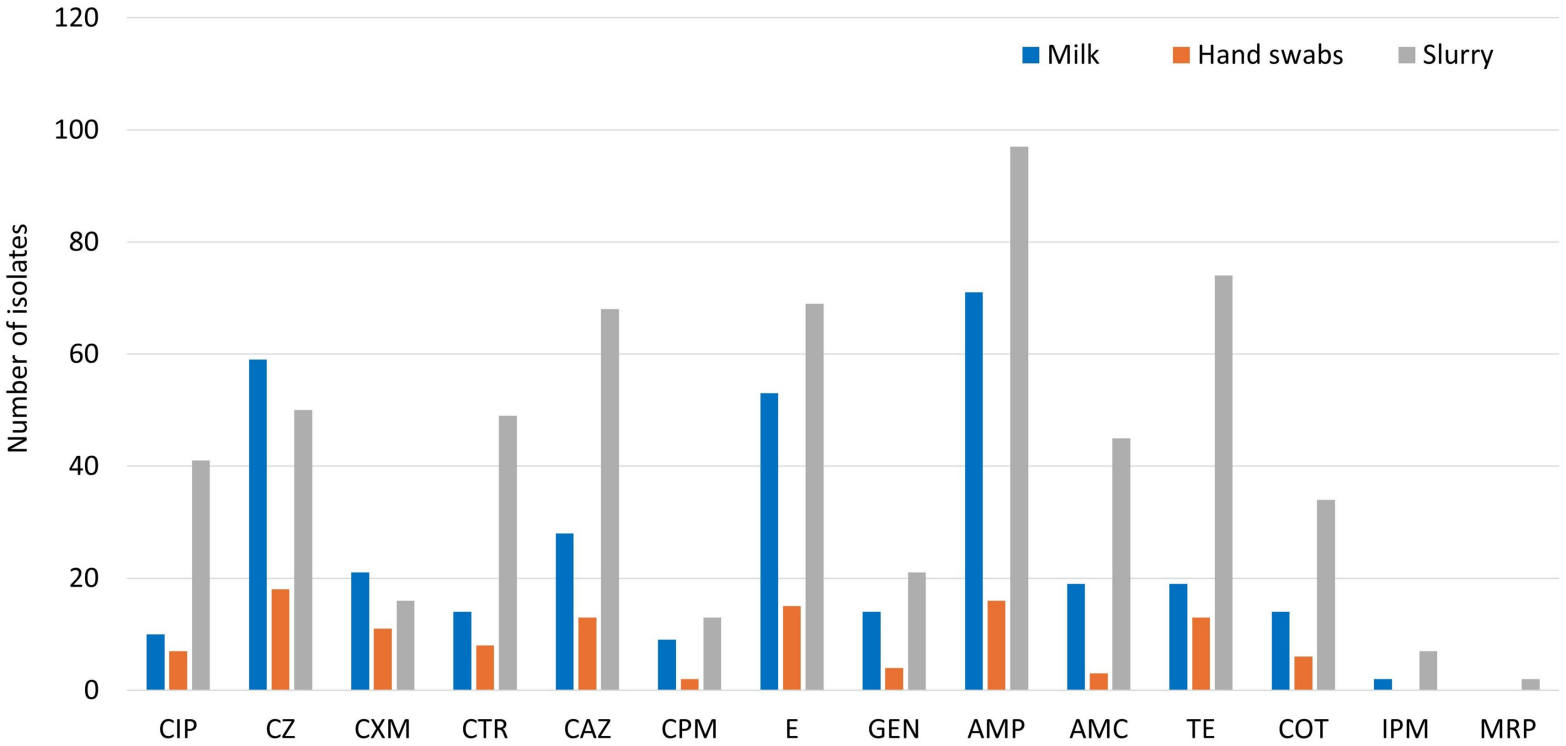
Phenotypic resistance profiling of *E. coli* isolates (CIP: Ciprofloxacin, CZ: Cefazolin, CXM: Cefuroxime, CTR: Ceftriaxone, CAZ: Ceftazidime, CPM: Cefepime, E: Erythromycin, GEN: Gentamicin, AMP: Ampicillin, AMC: Amoxicillin-Clavulanic acid, TE: Tetracycline, COT: Co-trimoxazole, IPM: Imipenem, MRP: Meropenem).

Among the recovered 139 *E. coli* isolates from milk samples subjected to Kirby-Bauer disk diffusion method, a high resistance observed against ampicillin (51.1%), followed by cefazolin (42.5%), and erythromycin (38.1%). Resistance to third- and higher-generation cephalosporins was detected in 36.7% of isolates, with ceftriaxone resistance at 10.1%, ceftazidime at 20.1%, and cefepime at 6.5%. Resistance to ciprofloxacin was observed in 7.2% of isolates, while 13.7% displayed resistance to tetracycline (Figure 1). Notably, 24.4% of isolates were classified as MDR, and 28.8% had a MAR index greater than 0.2 (Table 2). The presence of ESBL was confirmed in 28.1% of isolates through combined disc diffusion assay, indicated by an increase of ≥5 mm in the zone of inhibition with cefotaxime/clavulanic acid or ceftazidime/clavulanic acid discs compared to the antibiotics alone.

**Table 2:** Multi-antibiotic resistance (MAR) patterns of *E. coli* isolates.

Isolates’ sources	MDR (resistant to >3 classes of antibiotics)	MAR index (>0.2)	Resistance to all antibiotics	Resistance >10 antibiotics	Resistance 7–10 antibiotics	Resistance 5–7 antibiotics	Resistance 3–5 antibiotics	Resistance to <3 antibiotics	Sensitive to all antibiotics
Raw milk	34/139 (24.4%)	40/139 (28.7%)	0	2	4	10	24	84	15
Hand Swabs	15/37 (40.5%)	18/37 (48.6%)	0	1	3	5	9	12	7
Slurry	158/261 (60.5%)	187/261 (71.7%)	0	6	16	60	105	59	15

**Figure 2:**
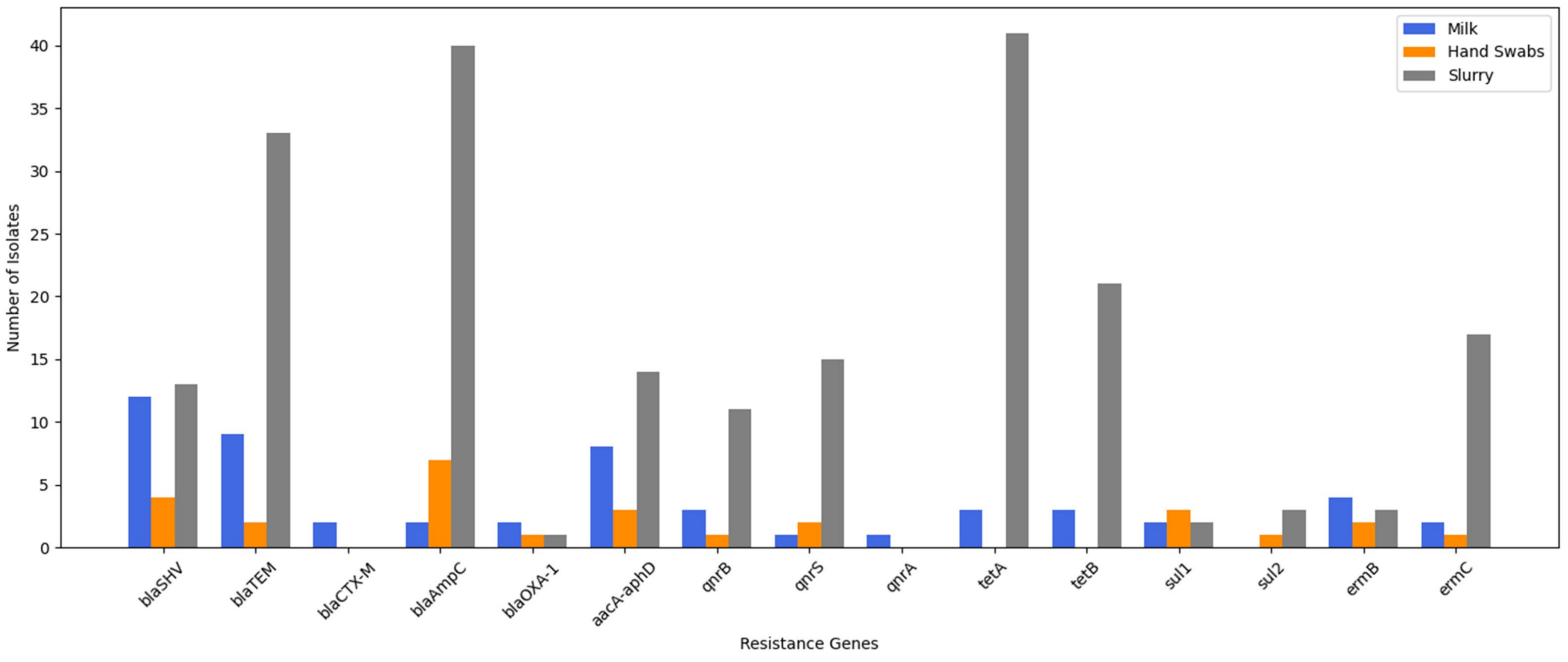
Genotypic resistance profiling of *E. coli* isolates.

The molecular characterization revealed that 19.3% of the isolates carried *β*-lactamase (ESBL/AmpC β-lactamase) genes. Among these, the *blaSHV* gene was identified in 8.6%, *blaTEM* in 6.5%, and *blaCTX-M*, *blaAmpC*, and *blaOXA-1* in 1.4% each. Also, the gentamicin resistance was conferred by the *aacA-aphD* gene in 5.8% of isolates, quinolone resistance genes were detected in 3.6% of isolates [*qnrB* (2.2%) and *qnrS* and *qnrA* (0.7% each)], tetracycline resistance genes were present in 4.4% of isolates [*tetA* and *tetB* (2.2% each)], sulfonamide resistance was attributed to the *sul1* gene in 1.4% of isolates, while erythromycin resistance genes (*ermB*) were found in 4.3% (Figure 2).

#### 3.2.2 *E. coli* isolates recovered from hand swabs of animal handlers

From the 37 *E. coli* isolates recovered from hand swabs of animal handlers, a high resistance was observed against cefazolin (48.7%), ampicillin (43.2%), and erythromycin (40.5%). Resistance to ceftazidime and tetracycline was seen in 35.1% of isolates each. Resistance to third- and higher-generation cephalosporins was identified in 62.2% of isolates, with ceftazidime resistance at 35.1%, ceftriaxone at 21.6%, and cefepime at 5.4% (Figure 1). Ciprofloxacin resistance was detected in 18.9% of isolates. Multidrug resistance was noted in 40.5% of isolates, while 48.7% exhibited a MAR index >0.2 (Table 2). ESBL production was confirmed in 45.9% of isolates through combination disc diffusion testing.

Molecular characterization showed that 37.8% of isolates carried *β*-lactamase (ESBL/AmpC β-lactamase) genes, with *blaAmpC* found in 18.9%, *blaSHV* in 10.8%, *blaTEM* in 5.4% and *blaOXA-1* in 2.7%. No isolates carried the *blaCTX-M* gene. Sulfonamide resistance genes, *sul1* were present in 8.1% and *sul2* was present in 2.7% of the isolates. Erythromycin resistance genes (*ermB* and *ermC*) were present in 8.1% of isolates, and gentamicin resistance (*aacA-aphD*) and quinolone resistance genes (*qnrB* and *qnrS*) were also detected in 8.1% of isolates each (Figure 2).

#### 3.2.3 *E. coli* isolates recovered from dairy herd slurry

Of the 261 *E. coli* isolates obtained from dairy herd slurry, 49.9% demonstrated resistance to third- and higher-generation cephalosporins, including ceftazidime (26.1%), ceftriaxone (18.8%), and cefepime (5.0%). Resistance to ampicillin was found in 37.2%, followed by tetracycline (28.4%), erythromycin (26.4%), cefazolin (19.2%), and amoxicillin-clavulanic acid (17.2%) (Figure 1). Ciprofloxacin resistance was observed in 15.7% of isolates. Multidrug resistance was detected in 60.5% of isolates, and 71.7% had a MAR index >0.2 (Table 2). ESBL production was identified in 38.7% of isolates through combination disc diffusion testing.

Molecular characterization revealed *β*-lactamase (ESBL/AmpC β-lactamase) genes in 33.3% of isolates, including *blaAmpC* in 15.3%, *blaTEM* in 12.6%, *blaSHV* in 4.9%, and *blaOXA-1* in 0.4%. None of isolate was found to harbour *blaCTX-M* gene. Further, 23.8% of isolates carried tetracycline resistance genes (*tetA* in 15.7%, *tetB* in 8.0%). Quinolone resistance genes (*qnrS* in 5.7%, *qnrB* in 4.2%) were present in 10.7% of isolates. Erythromycin resistance genes (*ermC* in 6.5%, *ermB* in 1.2%) and gentamicin resistance (*aacA-aphD* in 5.4%) were also observed. Sulfonamide resistance (*sul1 and sul2*) was detected in 1.9% of isolates (Figure 2).

### 3.3 Agreement between phenotypic and genotypic characterization of ESBL *E. coli*

The agreement between phenotypic and genotypic detection methods was assessed using Cohen’s Kappa coefficient, which revealed substantial agreement for milk isolates (*κ* = 0.720), and moderate agreement for both hand swabs (*κ* = 0.431) and dairy herd slurry samples (κ = 0.516) across the sample sources (Table 3).

**Table 3:** Phenotypic and genotypic detection of ESBL-*E. coli* across sample sources.

Sample source	Total isolates	Phenotypic detection	Genotypic detection (%)	Cohen’s Kappa	*blaSHV*	*blaTEM*	*blaCTX-M*	*blaOXA-1*
Milk	139	28.1% (*n* = 39)	18.0% (n = 25)	0.76	12	9	2	2
Hand Swabs	37	45.9% (*n* = 17)	18.9% (n = 7)	0.43	4	2	0	1
Slurry	261	38.7% (*n* = 101)	18.0% (n = 47)	0.51	13	33	0	1

### 3.4 Diversity and distribution of *β*-lactamase (ESBL/AmpC β-lactamase) genes across sample sources

**Figure 3:**
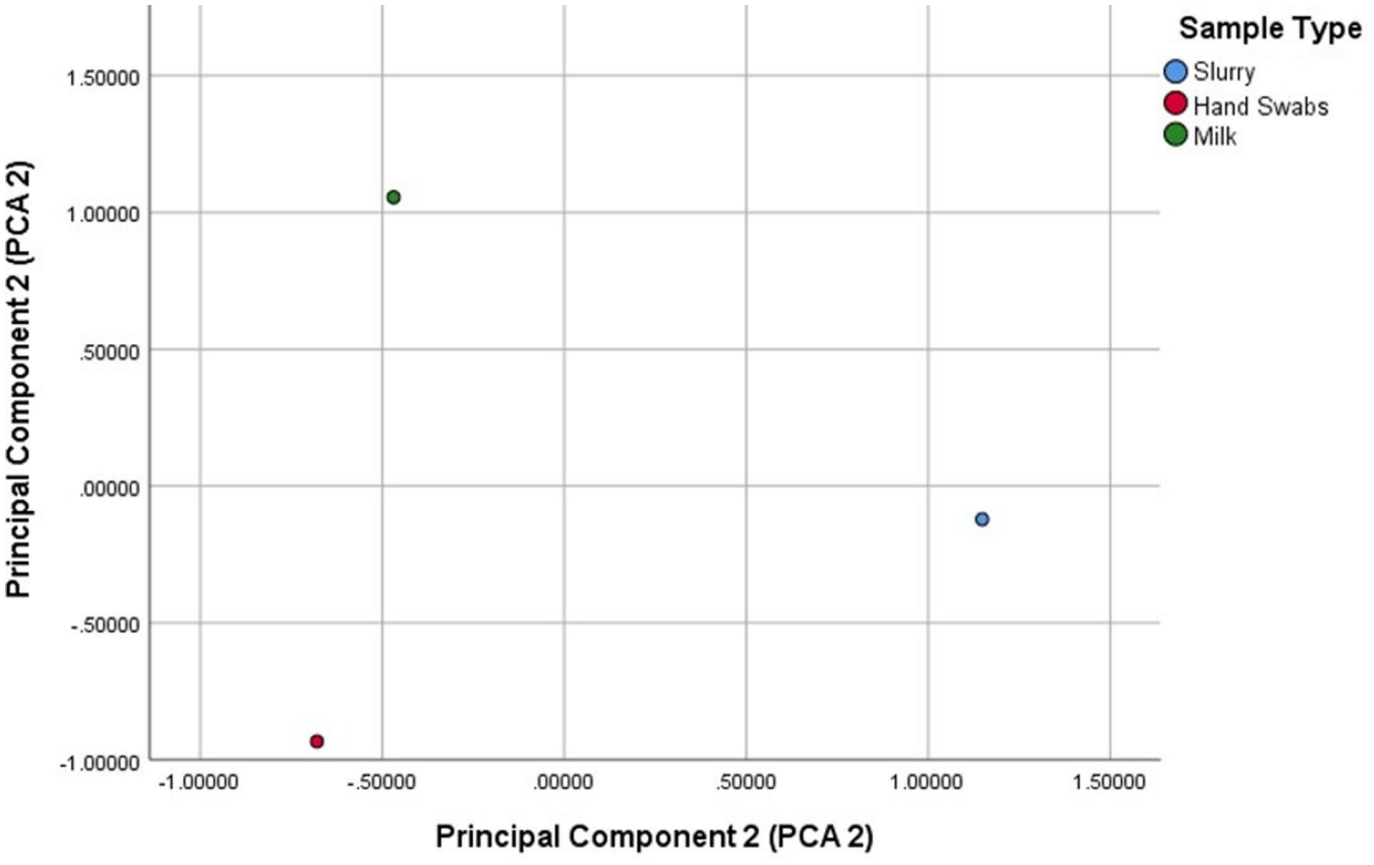
Principal component analysis (PCA) of variance of β-lactamase (ESBL/AmpC β-lactamase) gene distribution across sample sources.

The principal component analysis identified two principal components explaining 78.4% of the variance (Figure 3). PCA1 (50.2%) was strongly influenced by *blaTEM* and *blaAmpC*, while PCA2 (28.2%) was associated with *blaSHV* and *blaOXA-1*. The scatter plot revealed distinct clustering of sample types, with milk samples positioned higher on PCA2, reflecting contributions from *blaSHV* and *blaTEM*. Hand swab samples clustered lower on PCA1, dominated by *blaAmpC*, while dairy herd slurry samples were positioned higher on PCA1, driven by *blaAmpC* and *blaTEM*.

**Figure 4:**
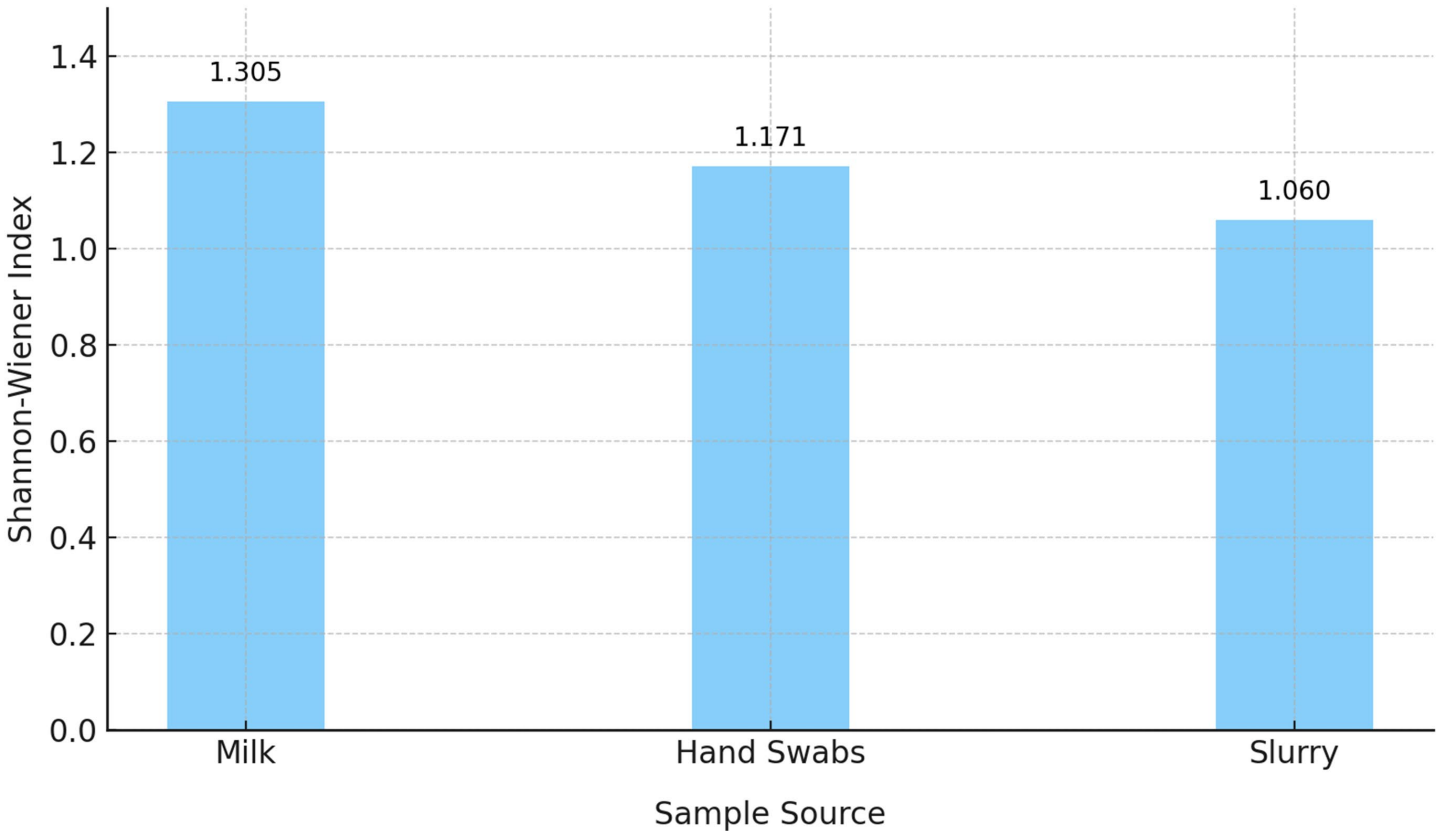
Shannon-Wiener diversity index of β-lactamase (ESBL/AmpC β-lactamase) genes across sample sources.

The Shannon-Wiener Diversity Index revealed notable variations in the diversity and distribution of *β*-lactamase genes across the three sample sources (Figure 4). Milk samples displayed the highest diversity (H′ = 1.305), with a broad representation of genes. Although *blaSHV* and *blaTEM* were dominant, the presence of *blaCTX-M*, *blaAmpC*, and *blaOXA-1* also contributed to the overall diversity. Hand swabs exhibited moderate diversity (H′ = 1.171), with a strong predominance of *blaAmpC*. Dairy herd slurry samples showed the lowest diversity (H′ = 1.060), largely driven by the dominance of *blaAmpC* and *blaTEM*.

### 3.5 Association between herd size, milk hygiene scores and *E. coli* occurrence

The logistic regression analysis showed that the ‘herd size’ had a negative but non-significant association with *E. coli* occurrence (*β* = −0.002, *p* = 0.298, OR = 0.99, 95%CI: 0.993, 1.002), whereas ‘milk hygiene score’ demonstrated a significant protective effect against *E. coli* occurrence (*β* = −1.672, *p* = 0.000, OR = 0.18, 95%CI: 0.13, 0.26). The Area Under the Curve (AUC) of the Receiver Operating Characteristic (ROC) curve was 0.89, indicating excellent discriminatory ability of the model.

### 3.6 Association between herd size, milk hygiene scores and multidrug resistant *E. coli* isolates

The results of linear regression to examine the effects of ‘herd size’ and ‘milk hygiene score’ on MAR index indicated that ‘herd size’ had significant negative effect on MAR index (*β* = −0.0004, *p* = 0.024, 95% CI: −0.001, 0.000). The ‘milk hygiene score’ had a statistically significant negative association with MAR index (*β* = −0.0203, *p* = 0.02, 95% CI: −0.038, −0.003). The overall model was significant (*p* < 0.001), with an R-squared value of 0.037.

### 3.7 Biofilm assay of the *E. coli* isolates

**Figure 5:**
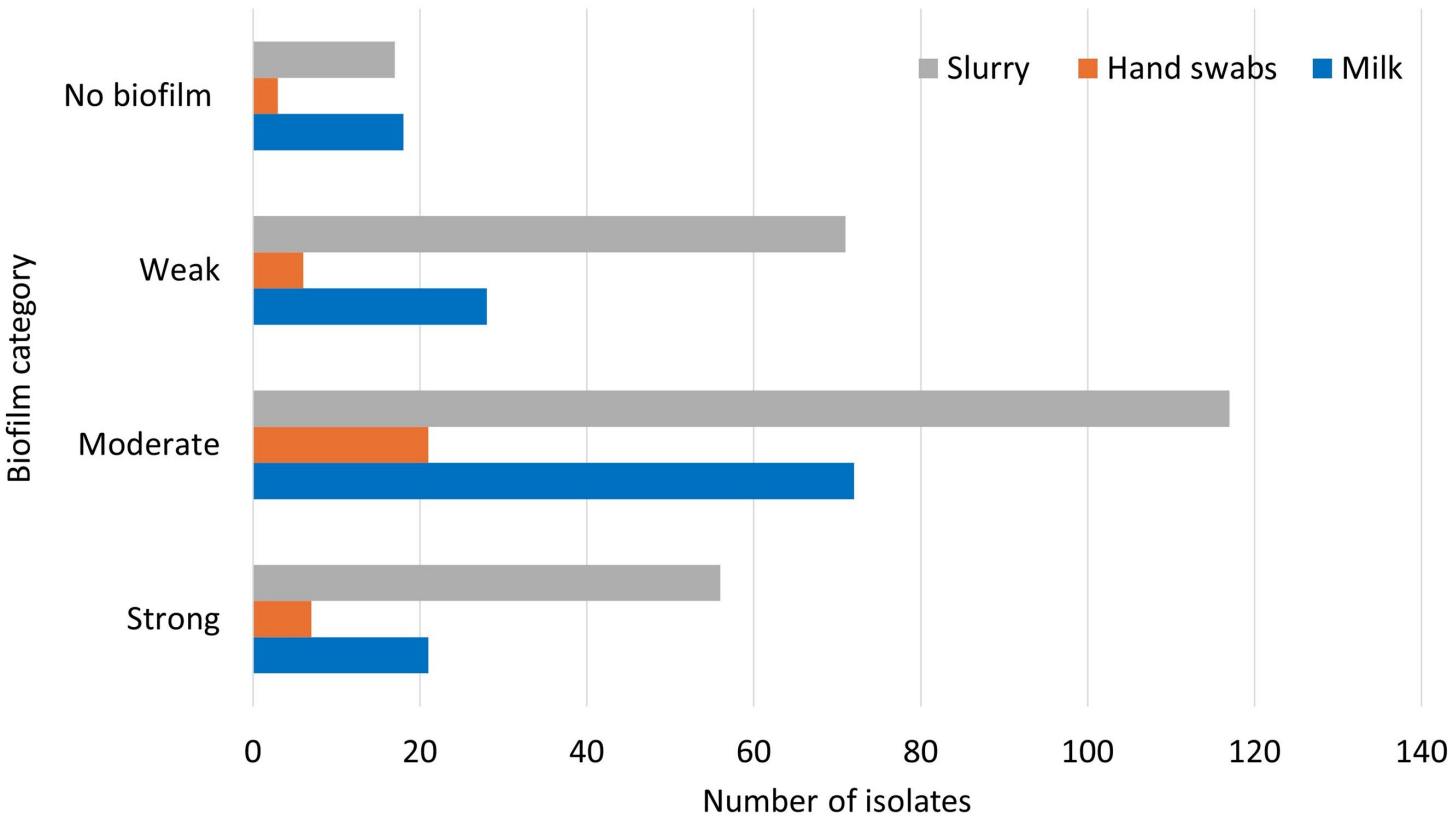
Biofilm formation of *E. coli* isolates from various sources.

Among the total 437 *E. coli* isolates recovered from various sources in the present study, 48.1% (210/437) were categorized as moderate biofilm producers, 24.0% (105/437) as weak biofilm producers, 19.2% (84/437) as strong biofilm producers and 8.7% (38/437) were non-biofilm producers. The classification of the biofilm forming ability of the *E. coli* isolates with respect to the source of samples is presented in Figure 5.

Among the milk *E. coli* isolates, 51.8% were found to be moderate biofilm producers (72/139), 20.1% were weak biofilm producers (28/139), 15.1% were high biofilm producers (21/139), and 12.9% (18/139) were found to be non-biofilm producers. Of the isolates from hand swabs of animal handlers, 56.8% were moderate biofilm producers (21/37), 18.9% were strong biofilm producers (7/37), 16.2% were weak biofilm producers (6/37) and 8.1% were found to be non- biofilm forming (3/37). Of the *E. coli* isolates from dairy herd slurry samples, 44.8% (117/261) were moderate biofilm producers, 27.2% (71/261) were weak biofilm producers, 21.5% (56/261) were strong biofilm producers and 6.5% (17/261) were found to be non-biofilm forming.

### 3.8 Association between biofilm formation and multidrug resistance of the recovered *E. coli* isolates

The MDR isolates of *E. coli* from three different sources: milk (*n* = 34), hand swab of animal handlers (*n* = 15) and dairy herd slurry samples (*n* = 158) were analyzed for their biofilm forming ability. Of the MDR isolates from milk samples, the majority (61.8%, *n* = 21) were moderate biofilm producers and 23.5% (*n* = 8) were strong biofilm forming isolates. Among the MDR isolates from the hand swabs, 60% (*n* = 9) were moderate biofilm producers and 20% (*n* = 3) were strong biofilm forming. The MDR isolates from dairy herd slurry samples were mainly moderate biofilm formers (48.7%, *n* = 77) and 24.1% (*n* = 38) were strong biofilm forming isolates. The classification of the biofilm forming ability of the MDR *E. coli* isolates with respect to the source of samples is presented in Table 4.

**Table 4:** Biofilm formation of MDR *E. coli* isolates from various sources.

Biofilm category	Type of sample		
	Bovine milk (*n* = 34)	Hand swab (*n* = 15)	Slurry (*n* = 158)
Strong	8 (23.5%)	3 (20.0%)	38 (24.1%)
Moderate	21 (61.8%)	9 (60.0%)	77 (48.7%)
Weak	5 (14.7%)	3 (20.0%)	43 (27.2%)

**Figure 6:**
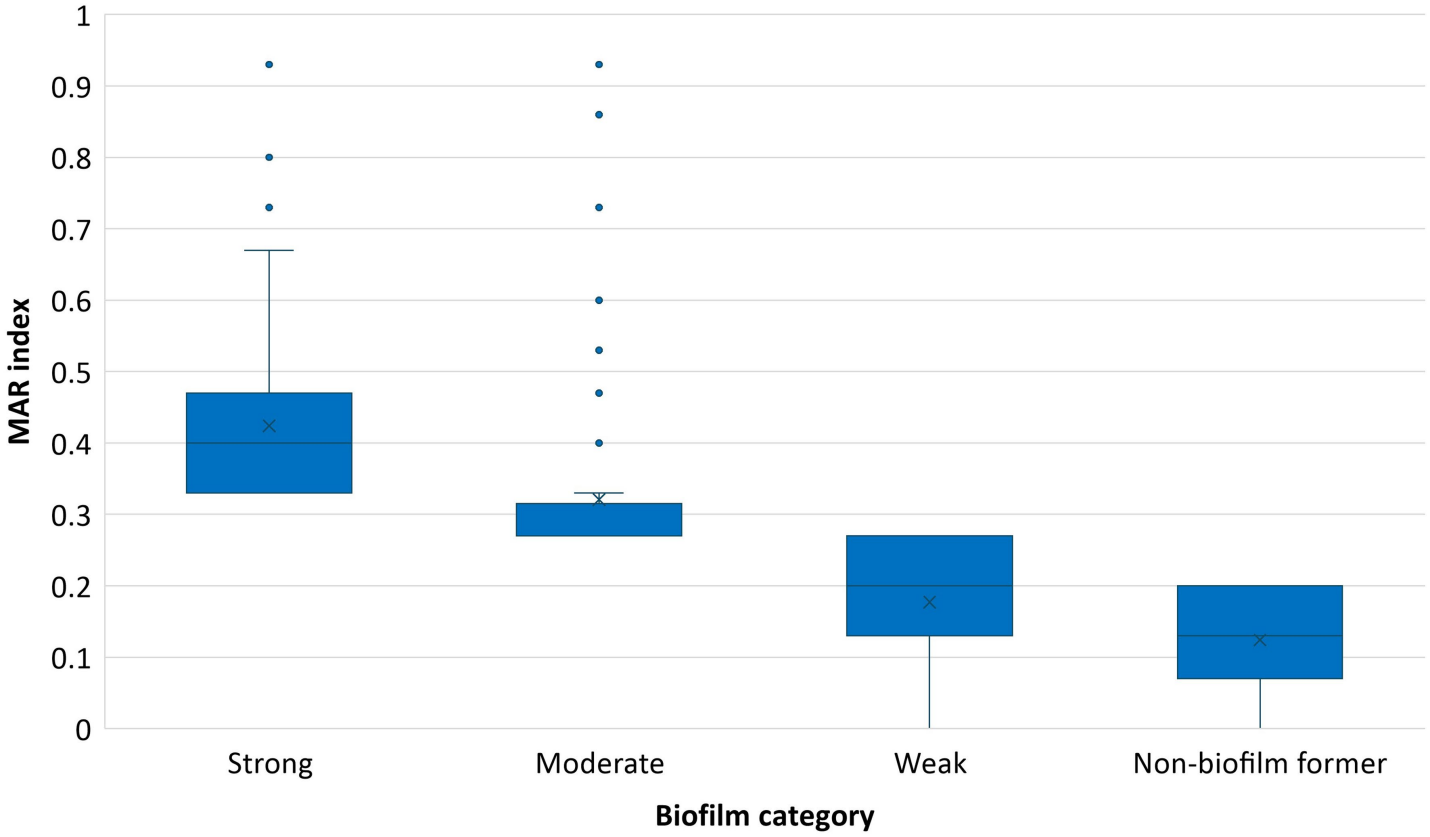
Association between MAR index and biofilm forming potential of *E. coli* isolates.

A strong association (*p* value < 0.01) between biofilm formation potential and MAR index was observed in the *E. coli* isolates. The isolates within the category of ‘strong’ biofilm formers were more likely to exhibit higher MAR index values, suggesting a potential link between biofilm formation capability and antibiotic resistance. The box plot also reveals the increase in MAR index across the biofilm formation categories (Figure 6).

In case of MDR *E. coli* isolates from various sources, no significant difference was found between the strong biofilm forming ability of the isolates, from milk and hand swabs (z value: 0.27; p value: 0.78); milk and slurry (z value: −0.06, p value: 0.95); hand swab and slurry (z value: −0.35, p value: 0.73).

## 4 Discussion

The dairy farm environment can serve as a potential hotspot for antibiotic resistance gene(s) exchange and spread, as the presence of antibiotics, nutrients, and bacterial populations fosters the development of multidrug-resistant bacteria, posing a significant public health threat (36). As both a commensal and opportunistic pathogen, *E. coli* is widely recognized as an important indicator organism for monitoring AMR due to its ubiquitous presence and capacity to acquire resistance determinants (37, 38). It plays a critical role in tracking clinically significant antibiotic-resistant phenotypes, such as resistance to extended-spectrum beta-lactams, across human, animal, and environmental samples (39).

The present study highlights the prevalence of *E. coli* contamination in dairy farms in Punjab, with 34.3% (139/405) of milk samples, 9.1% (37/405) of hand swabs of animal handlers, and 64.4% (261/405) of slurry samples testing positive. These findings are consistent with global reports, such as studies from dairy farms in Zambia (51.2%) (40), China (34.3%) (12), and Egypt, where the prevalence of *E. coli* in raw milk ranges from 20 to 76.4% (11, 41). Similarly, studies from India report *E. coli* contamination in raw milk ranging from 40 to 60% (28, 42, 43). In present study, the median milk hygiene score was observed as 3 (out of 5), indicating moderate adherence to hygiene practices. However, key components, such as regular screening for mastitis and prompt removal of dung from the dairy environment, scored particularly low. This suggests that deficiencies in these hygiene practices not only compromise milk safety but also create favorable conditions for the proliferation and spread of pathogens within the dairy environment. Further, the logistic regression analysis between herd size, milk hygiene score and *E. coli* occurrence highlights the role of milk hygiene in reducing *E. coli* occurrence, as evidenced by the significant protective effect of higher hygiene scores, while herd size showed no significant association, underscoring the importance of prioritizing hygiene practices in controlling contamination (44). These findings reinforce the necessity for targeted training and awareness programs for dairy workers, alongside stringent enforcement of hygiene protocols, to minimize contamination and improve milk quality in dairy farms.

Previous studies have suggested that raw milk may act as a potential route for the transmission of resistant pathogens to humans (40, 45). In the present study, 19.3% of the isolates were genotypically confirmed as *β*-lactamase (ESBL/AmpC β-lactamase) producers. The prevalence of ESBL *E. coli* has been reported from raw milk ranging from 7.91% in India (46) to 19.5% in dairy farms of China (47). Recent studies have indicated increasing prevalence of multidrug resistant β-lactamase producing *E. coli* in raw milk (40, 42, 48). Around 24.4% of the isolates from raw milk in the present study were MDR. In a recent study from Indonesia 7.26% of the isolates were MDR (49). The findings underscore the presence of multidrug-resistant *E. coli* in raw milk, pointing to the need for enhanced hygiene measures and responsible antibiotic use to curb AMR. It is important to note that while *E. coli* serves as a key indicator organism for antimicrobial resistance surveillance, the isolates examined in this study were not screened for virulence-associated genes. As such, the findings should be interpreted as representative of AMR trends in general *E. coli* populations from diverse sources in the dairy farm interface rather than pathogenic strains.

Occupational exposure among dairy workers further exacerbates the risk, as evidenced by studies from Egypt where *E. coli* was detected in 20% of hand swabs from dairy handlers (14), and 12.5% of dairy farm workers carried ESBL *E. coli* in China (50). This is in concordance with present study where 40.5% of the *E. coli* isolates from hand swabs were multidrug resistant and 37.8% were *β*-lactamase producers, highlighting the occupational health risk posed by resistant strains, which can colonize workers and contribute to community-level AMR dissemination.

The *E. coli* isolates from dairy slurry in this study exhibited substantial AMR with 49.9% resistant to third- and higher-generation cephalosporins. Resistance to ampicillin (37.2%), tetracycline (28.4%), erythromycin (26.4%), cefazolin (19.2%), and amoxicillin-clavulanic acid (17.2%) highlights the widespread nature of AMR in dairy slurry, as evidenced by other researchers in the region (27). These findings are also consistent with previous studies that identified high levels of AMR in agricultural settings highlighting the possible role of dairy manure in AMR dissemination (12, 51, 52). Recent studies emphasized that the persistence of mobile genetic elements, such as integrons and plasmids in manure-treated soils, significantly contribute to the dissemination of AMR genes (16, 18). These results emphasize the critical need for stringent antibiotic stewardship, effective dairy waste management, and routine AMR surveillance in dairy farms to mitigate the risk of AMR.

The results of the present study demonstrated substantial agreement between phenotypic and genotypic ESBL detection in milk samples (*κ* = 0.720), suggesting reasonable concordance between the two methods, with some discrepancies likely attributable to non-ESBL *β*-lactamase activity or limitations in gene detection. Moderate agreement was observed for both hand swabs (κ = 0.431) and dairy herd slurry samples (κ = 0.516), indicating greater variability in detection outcomes. These findings suggest that phenotypic methods, while valuable for routine screening, may not consistently reflect the underlying genotypic profile, particularly in more heterogeneous or environmentally exposed sample sources such as slurry.

The PCA and diversity indices revealed distinct *β*-lactamase gene profiles across sample sources. The higher diversity of β-lactamase (ESBL/AmpC β-lactamase) genes observed in milk samples may be attributed to the convergence of multiple contamination sources during milk collection and handling at dairy farms. Unlike slurry or hand swab samples, which primarily reflect the microbial populations of a single reservoir, milk can act as a common interface for bacteria originating from various sources, including the udder, milking equipment, animal handlers, and the farm environment. This mixing facilitates the introduction and coexistence of diverse β-lactamase producing *E. coli* strains, each potentially harbouring different resistance genes. Conversely, hand swabs exhibited moderate diversity, dominated by *blaAmpC*, likely reflecting selective pressure or repeated contamination from human–animal contact. Further, dairy herd slurry samples showed the lowest diversity, primarily due to the dominance of *blaAmpC* and *blaTEM*, suggesting their role as an environmental reservoir and potential hotspot for resistance gene amplification.

The *blaAmpC* gene was the most prevalent *β*-lactamase gene in the study, followed closely by *blaTEM*. These findings align with earlier reports, such as Yang et al. (18), who observed high prevalence of ESBL genes (*blaTEM-1*, *blaOXA-1*) and AmpC β-lactamase genes (*blaAmpC*) in intensive dairy farms of China. Also, a high prevalence of the *blaTEM* genes was reported in a study from South India, where Joseph and Kalyanikutty (53) reported its detection in 42.85% of the isolates. Similarly, a study conducted in the dairy farm environment in China found that 56.3% of beta-lactam–resistant isolates harbored *blaTEM* genes (54). The identification of such high-priority antimicrobial resistance genes, particularly those conferring resistance to extended-spectrum cephalosporins (e.g., *blaTEM, blaAmpC etc*) in *E. coli* from dairy farm environments highlights critical public health and economic challenges (54, 55). Although this study confirms the presence of β-lactamase and other resistance genes in *E. coli* isolates, we did not investigate whether these genes are located on plasmids or other mobile genetic elements. This is a key limitation, as plasmid-borne resistance genes can facilitate horizontal gene transfer across bacterial populations. Future studies employing whole-genome sequencing or plasmid profiling are essential to understand the mobility and dissemination potential of these resistance determinants.

Around 47.4% of the isolates of the present study were multidrug resistant in concordance with study from dairy farm environment in China where 44.4% of the isolates were multidrug resistant (54). The linear regression analyses demonstrated that both herd size and milk hygiene score were significantly and negatively associated with the MAR index, indicating that larger herd sizes and improved milk hygiene are linked to reduced multiple antibiotic resistance among *E. coli* isolates. However, the minimal effect size for herd size (*β* = −0.0004) and the low R-squared value (0.037) suggested that these two variables explain only a small portion of the variation in MAR index, highlighting the potential influence of other unmeasured factors such as antibiotic usage patterns, environmental conditions, or broader management practices in influencing antimicrobial resistance levels (56–58). Notably, while linear regression identified both variables as significant predictors of MAR index, logistic regression analysis found that only milk hygiene score had a significant protective effect against *E. coli* occurrence, with herd size showing no significant association. These findings collectively emphasize the central importance of milk hygiene in mitigating antimicrobial resistance and reducing *E. coli* contamination, reinforcing that prioritizing improved hygiene practices is effective for controlling microbial risks in dairy farm environments.

Many studies across the globe have reported a positive correlation between biofilm formation ability and resistance to antibiotics in *E. coli* strains which align with the current study’s observation of a strong association (*p* < 0.01) between biofilm formation potential and the MAR index of *E. coli* isolates (42, 59). This association can be attributed to the protective environment biofilms provide enhancing bacterial survival under adverse conditions including antibiotic exposure, by acting as a barrier to antibiotic penetration and facilitating horizontal gene transfer of resistance determinants (23). These findings underscore the dual threat posed by biofilm-forming *E. coli* in dairy environments, their enhanced persistence under antimicrobial pressure and their contribution to the spread of resistance. Addressing biofilm formation is therefore critical for mitigating AMR in dairy farms and safeguarding public health.

Although direct data on antimicrobial usage (AMU) were not collected in this study, existing literature indicates significant AMU in Punjab’s dairy sector. A recent quantitative analysis reported that antibiotics such as enrofloxacin, ceftriaxone, and amoxicillin are commonly used (57, 58). Furthermore, qualitative studies have identified factors contributing to injudicious antibiotic use, including self-treatment by farmers, over-the-counter availability of antibiotics, and limited access to veterinary services (57, 58, 60, 61). These practices likely contribute to the AMR patterns observed in our study.

A study by Wu et al. (22) highlighted that resistance mechanisms often overlap between agricultural and human clinical settings, with plasmid-mediated ARGs acting as a key driver of resistance spread. In a study from Nigeria, human and environmental *E. coli* isolates from the same poultry farm were found to exhibit similar antimicrobial resistance genes and plasmid replicon profiles (62). Such evidence underscores the critical importance of integrated surveillance systems using a ‘One Health’ approach to monitor and address the emergence and spread of AMR (19). Comprehensive studies incorporating epidemiological, genomic, and ecological analyses are necessary to trace the origins and pathways of resistant strains and implement evidence-based strategies to mitigate their impact.

Multidrug resistant *E. coli* poses a critical public health threat due to their dual role as pathogens and carriers of transferable resistance genes, emphasizing the need for integrated One Health surveillance to curb the spread of antimicrobial resistance across human, animal, and environmental domains. The increasing global trend of AMR necessitates urgent action, particularly in regions with intensive livestock farming (63). Policymaking efforts must prioritize antibiotic stewardship, improved farm management practices, and stringent controls on the use of antibiotics in animal husbandry (64).

Further, advances in metagenomics and whole-genome sequencing have provided powerful tools to uncover resistance pathways and monitor the spread of ARGs across reservoirs. However, challenges remain in translating scientific findings into policy actions, particularly in resource-limited settings. Encouraging public-private partnerships and strengthening local capacities for AMR monitoring can foster sustainable strategies to combat this growing threat. As underscored by the present study, continuous monitoring of AMR and its genetic determinants is critical for safeguarding public health and ensuring the sustainability of agricultural practices.

## 5 Study limitations

The present study has certain limitations. Initially, we selected the sampling districts randomly and attempted to select farms in a random manner. Due to the large sample size and the need to obtain agreement for enrolment, we had to resort to convenience sampling, whenever required. However, the study’s stratified design and proportional representation across agro-climatic zones and farm types strengthen the external validity of the findings. Nevertheless, some degree of selection bias cannot be excluded, and future studies should aim for full randomization where feasible.

Another limitation is the lack of characterization of *E. coli* into pathotypes, as the primary objective of this study was to assess *E. coli* as a surveillance indicator for AMR in the dairy environment. Additionally, the study did not target the full set of *β*-lactamase genes but focused on the commonly reported genes in the region, aligning with the study’s mandate.

A further limitation is the absence of direct data on antimicrobial usage (AMU) at the farm level, which is a well-recognized driver of AMR. The inclusion of AMU data could have provided a more comprehensive understanding of resistance dynamics and enhanced the explanatory power of the statistical models. However, due to the cross-sectional design of the study, it was not feasible to collect reliable longitudinal AMU data, which typically requires repeated monitoring over time.

## 6 Conclusion

This study highlights the significant prevalence of *E. coli* in dairy environments, with substantial contamination observed in milk, hand swabs, and slurry, posing notable public health risks. The good milk hygiene practices were strongly associated with reduced *E. coli* prevalence and lower MAR index values, underscoring the importance of hygiene practices in mitigating AMR. The dairy herd slurry was identified as a major reservoir for resistance genes, emphasizing the critical need for effective waste management practices to limit AMR dissemination. The strong association between biofilm formation potential and the MAR index in *E. coli* underscores the critical role of biofilms in enhancing antimicrobial resistance, highlighting the need to target biofilm formation to mitigate AMR in dairy farms. The study underscores the need for integrative strategies that align with the principles of One Health, addressing the interconnected challenges of AMR in a holistic manner at the dairy environment interface.

## Data Availability

The raw data supporting the conclusions of this article will be made available by the authors, without undue reservation.
